# Population-Scale Pharmacogenomic Profiling in the Western Balkan Populations

**DOI:** 10.3390/ph19101526

**Published:** 2026-09-26

**Authors:** Isidora Ćurić, Đorđe Pavlović, Marina Jelovac, Biljana Stanković, Nikola Kotur, Vladimir Gašić, Bojan Ristivojević, Nataša Tošić, Jelica Predojević-Samardžić, Marija Vukovic, Jelena Roganovic, Violeta Anastasovska, Olivera Miljanovic, George P. Patrinos, Kariofyllis Karamperis, Christina Mitropoulou, Vita Dolzan, Marianna Lucafò, Gabriele Stocco, Sonja Pavlović, Branka Zukić

**Affiliations:** 1 Group for Molecular Biomedicine, Institute of Molecular Genetics and Genetic Engineering, University of Belgrade, 11042 Belgrade, Serbia; isidora.curic@imgge.bg.ac.rs (I.Ć.); djordje.pavlovic@imgge.bg.ac.rs (Đ.P.); marina.jelovac@imgge.bg.ac.rs (M.J.); biljana.stankovic@imgge.bg.ac.rs (B.S.); nikola.kotur@imgge.bg.ac.rs (N.K.); vladimir.gasic@imgge.bg.ac.rs (V.G.); bojan.ristivojevic@imgge.bg.ac.rs (B.R.); natasa.tosic@imgge.bg.ac.rs (N.T.); sonja.pavlovic99@gmail.com (S.P.); 2 Paediatric Clinic, University Clinical Centre of the Republic of Srpska, 78000 Banja Luka, Bosnia and Herzegovina; predojevicjelica5@gmail.com; 3 Institute of Clinical Pathology, University Clinical Centre of the Republic of Srpska, 78000 Banja Luka, Bosnia and Herzegovina; marija.vukovic@kc-bl.com; 4 Division of Hematology and Oncology, Department of Pediatrics, Clinical Hospital Centre Rijeka, 51000 Rijeka, Croatia; jelena.roganovic02@gmail.com; 5 Department of Neonatal Screening, University Clinic for Pediatrics, Faculty of Medicine, Ss. Cyril and Methodius University in Skopje, 1000 Skopje, North Macedonia; violeta_anastasovska@yahoo.com; 6 Center for Genomic Medicine and Immunology, Clinical Center of Montenegro, 81000 Podgorica, Montenegro; oliveram@ucg.ac.me; 7 Department of Pharmacy, University of Patras, 26504 Patras, Greece; gpatrinos@upatras.gr (G.P.P.); k.karamperis@upatras.gr (K.K.); 8 Hellenic Pasteur Institute, 11521 Athens, Greece; 9 Big Data Research Institute, Vin University, Hanoi 100000, Vietnam; 10 Department of Genetics and Genomics, College of Medicine and Health Sciences, United Arab Emirates University, Al-Ain 15551, Abu Dhabi, United Arab Emirates; c.mitropoulou@goldenhelix.org; 11 The Golden Helix Foundation, London SE1 8RT, UK; 12 Pharmacogenetics Laboratory, Institute of Biochemistry and Molecular Genetics, Faculty of Medicine, University of Ljubljana, SI-1000 Ljubljana, Slovenia; vita.dolzan@mf.uni-lj.si; 13 Department of Medical, Surgical and Health Sciences, 34149 Trieste, Italy; mlucafo@units.it (M.L.); stoccog@units.it (G.S.)

**Keywords:** pharmacogenomics, Western Balkan population, star allele, whole genome sequencing, bioinformatics, personalized medicine, novel pharmacogenomic variants

## Abstract

**Background:** Pharmacogenomic variation contributes to interindividual differences in drug response, yet population-specific pharmacogenomic profiles remain insufficiently characterized in the Western Balkans (WB). This study aimed to comprehensively characterize pharmacogenomic variability in the WB populations using whole-genome sequencing (WGS), assess population differences in pharmacogenomic profiles, evaluate concordance among star allele calling tools, and identify rare and previously unreported variants in pharmacogenes. **Methods:** WGS data from 250 healthy individuals from five WB populations were analyzed across 88 pharmacogenes. Star alleles were assigned using Aldy4, PharmCAT, PyPGx, and Stargazer. Principal component analysis (PCA) was used to assess population structure, and pharmacogenomic allele and phenotype frequencies were compared within five European subpopulations. Unreported pharmacovariants were annotated using Ensembl VEP, gnomAD, ClinVar, and ClinPGx, with functional impact assessed using multiple in silico prediction tools. **Results:** A total of 357 star alleles were identified, with high tool concordance except for complex genes such as CYP2D6. WB populations clustered within the European genetic space, with limited subpopulation differentiation. Significant differences were observed within WB and compared with European populations, including clinically relevant genes CYP2B6, CYP2C19, RYR1, and VKORC1. Among 33 clinically relevant pharmacogenes, 22,638 SNVs were identified, including six previously unreported variants. **Conclusions:** The WB populations show substantial pharmacogenomic similarity to other European populations, while also exhibiting clinically relevant population-specific differences. Comprehensive WGS-based profiling revealed both established and previously unreported pharmacogenomic variation, highlighting the importance of including underrepresented populations in precision medicine research.

## 1. Introduction

Genetic variation plays a critical role in determining interindividual differences in drug response. To date, genomic variants affecting drug absorption, distribution, metabolism, and excretion (ADME), as well as drug targets and downstream pharmacodynamic pathways, have been implicated in variability in efficacy, toxicity, and overall therapeutic outcomes across a wide range of medications [1,2]. ClinPGx classifies pharmacogenomic (PGx) information in drug labels as required, recommended, actionable, and informative genetic testing, based on documentation from regulatory agencies including the U.S. Food and Drug Administration (FDA) and the European Medicines Agency (EMA) [3]. PGx thus represents a cornerstone of precision medicine, linking interindividual genomic variability with more informed prescribing decisions and improved treatment individualization [2,4]. It is important to stress that the clinical utility of PGx depends not only on the identification of relevant genetic variants but also on their standardized interpretation within clinically meaningful frameworks. In this context, recent efforts by the Clinical Genome Resource Pharmacogenomics Working Group (ClinGen PGxWG) and the Clinical Pharmacogenetics Implementation Consortium (CPIC) have emphasized the importance of harmonized approaches for defining gene–drug clinical validity, variant actionability, and allele functional status, thereby supporting broader and more consistent implementation of PGx in genomic medicine [5,6].

The advent of high-throughput sequencing technologies, particularly next-generation sequencing (NGS), has markedly enhanced the ability to identify genetic variation underlying drug response [7]. In particular, whole-genome sequencing (WGS) provides a comprehensive platform for pharmacogenomic analysis, enabling the detection of single-nucleotide variants, structural variation, copy number changes, and complex haplotypes across pharmacogenes, including loci that are difficult to resolve using more limited approaches [8]. This is especially relevant for highly polymorphic and structurally complex genes, where accurate haplotype and star allele assignment may be challenging. However, the large-scale data generated by WGS require sophisticated bioinformatics tools and computational approaches to translate raw genomic information into clinically actionable insights. In this context, the use of multiple complementary tools for star allele calling has been shown to improve accuracy and reliability, particularly for genes such as *CYP2D6*, for which algorithm-dependent discrepancies may arise due to structural complexity and differences in allele definition or calling strategy [9,10,11].

While general PGx guidelines provide a framework for therapy individualization, population-specific genetic characteristics may also be relevant [12,13]. Substantial variability in drug efficacy and the risk of adverse drug reactions has been observed across populations for the same therapeutic agents. These differences are largely driven by variation in the frequency and distribution of pharmacogenomic variants, including common, rare, and structurally complex alleles, highlighting the importance of population-tailored PGx profiling [14].

Despite the growing implementation of PGx in clinical practice, population-specific variability in pharmacogenes remains insufficiently characterized in many regions, including the Western Balkans (WB). Given the complex demographic history and genetic diversity of this region, a comprehensive understanding of pharmacogenomic variation is essential for the effective translation of precision medicine into clinical settings. Recent pharmacogenomic profiling of the Serbian (SRB) population has suggested that locally relevant PGx patterns may be obscured when regional cohorts are interpreted solely through broad European reference datasets [15]. Extending this work to a unified Western Balkan framework is therefore important not only for regional characterization, but also as a broader model for understanding how fine-scale population diversity may influence the implementation of pharmacogenomics across Europe.

Therefore, the present study aimed to systematically characterize the pharmacogenomic landscape of the WB populations using WGS data. Specifically, we sought to define the distribution of star alleles across clinically relevant pharmacogenes, assess interpopulation differences in allele and haplotype frequencies in comparison with European reference populations, and evaluate predicted pharmacogenomic phenotypes based on established CPIC guidelines. In addition, we investigated the population pharmacogenetic structure of the WB cohorts, evaluated concordance between multiple star allele calling tools, and identified rare and novel variants with potential functional and clinical significance. Through this integrative approach, this study provides a comprehensive framework for understanding pharmacogenomic variability in the WB population and supports its inclusion in global precision medicine initiatives.

## 2. Results

### 2.1. Inter-Tool Concordance and Consensus of PGx Calls

Genotype concordance was assessed using Aldy, PyPGx, PharmCAT, and Stargazer (for *CYP2D6*). Given that a consensus approach may improve accuracy and reduce false-positive calls, concordance was evaluated using a three-tool consensus model. Absolute concordance was observed for 18 genes (51.4%), with complete agreement across tools. High concordance (>90%) was observed for 28 genes (80%). Lower concordance was primarily associated with structurally complex genes or differences in allele definitions. Notably, reduced agreement was observed for *CYP2D6* (82.8% for Aldy 4 and PyPGx and 59.2% for Aldy4, PyPGx, and Stargazer), *CYP4F2* (84.8%), *DPYD* (71.6%), *RYR1* (72.4%) and *NAT1* (67.2%). For *UGT2B7* and *VKORC1*, the tools used different allele definitions; however, the resulting haplotype interpretations for main variants were comparable. In the case of *VKORC1*, although Aldy4 reported a variant other than rs9923231, only rs9923231 was considered in the present analysis. For *UGT2B7*, we compared Aldy4 and PyPGx genotyping results specifically for the UGT2B7*2 star allele, and both tools produced concordant results. PyPGx results were used for the final analysis because the 1000 Genome project data used for comparison were annotated using PyPGx, providing a consistent framework for allele annotation between the study and reference European datasets. Discordant calls were further examined using binary alignment map (BAM) files, and genotypes consistent with the underlying variant evidence were selected for downstream analyses (Table S3). The genotype concordance for each gene, as well as the source of the discrepancies, can be found in Table S3.

### 2.2. Population Structure Analysis

The principal components analysis (PCA) was initially performed on whole-genome data including all 1000 Genomes Project populations together with the WB samples as a quality control step, and yielded consistent population clustering patterns (Figure 1a). We then performed a PCA based on variants in pharmacogenes only, which similarly revealed clear separation of global populations (Figure 1b) along the first two principal components (PC1 and PC2). PC1 (43.2%) primarily distinguished African populations from non-African populations, while PC2 (19.9%) separated East Asian populations from European and admixed groups. The WB samples clustered within the European genetic space, showing closest proximity to the EUR populations indicating shared ancestry, with slight dispersion toward admixed American and South Asian groups, indicating minor genetic overlap. When restricted to European populations (Figure 2), PCA showed tighter clustering, with PC1 (11.3%) and PC2 (10.9%) capturing more subtle population structure differences. The WB samples overlapped extensively with IBS and TSI, while also showing proximity to CEU and GBR subpopulations. The FIN subpopulation formed a more distinct cluster, consistent with their known genetic differentiation within Europe. Overall, these results indicate that the WB populations are genetically consistent with European populations, with the closest similarity to Southern European (Iberian populations in Spain and Toscani in Italy) groups and no evidence of major population stratification. These findings are consistent with the observed pharmacogenomic profiles, where the WB population shows high concordance with European reference populations.

### 2.3. PGx of the Western Balkan Population

Star allele analyses were initially performed across 88 pharmacogenes (Table S4). Of these, in five genes in which only reference alleles were detected (*ACYP2*, *CACNA1S*, *CYP2R1*, *CYP17A1*, and *CYP26A1*) and five genes deviated from Hardy–Weinberg equilibrium (HWE) (*CYP2E1*, *G6PD*, *GSTT1*, *IFNL3*, and *SULT1A1*), all of which were excluded from comparative analyses, leaving 78 genes for within-WB comparisons (Table S4). For comparisons with European reference populations, MT-RNR1 was additionally excluded because reliable population-specific allele frequencies could not be obtained for this gene, resulting in a final set of 77 pharmacogenes (Table S4). Among the analyzed pharmacogenes, CYP2D6 was identified as the most polymorphic gene, with a total of 33 alternative haplotypes reported (Table S5). When considering the proportion of non-reference (alternative) haplotypes, the highest variability was observed in three genes: MTHFR (100%), CYP3A5 (99.96%), and CYP1B1 (99.90%) (Table S6). In contrast, the lowest variability was detected in G6PD (0.004%), NUDT15 (0.006%), CFTR (0.008%), UGT1A6 (0.008%), F2 (0.014%), F5 (0.014%), and TPMT (0.026%) (Table S6).

A total of 357 star alleles were identified across 88 pharmacogenes in the WB population. Allele frequencies were compared both within WB subpopulations and between each subpopulation and the combined dataset (Table S4). To account for multiple testing, a Bonferroni correction was applied (0.05/78), and statistical significance was defined as *p* < 0.0006. Significant differences in haplotype frequencies were observed for only one of the 78 analyzed pharmacogenes. Specifically, the frequency of one star allele in one pharmacogene differed significantly between the Bosnia&Herzegovina (B&H) and North Macedonian (NMC) subpopulations, with a higher frequency observed in B&H (GSTP1*B, *p* < 0.00053). A complete list of allele frequency comparisons and corresponding *p*-values is provided in Table S7. Where phenotype data were available (18 genes, frequency Table S8), phenotype frequencies were also compared across subpopulations. After Bonferroni correction (0.05/18; *p* < 0.0027), a significant difference was observed for CYP2B6, with the poor metabolizer phenotype being more frequent in the B&H subpopulation compared to other WB groups (*p* = 0.000137). Detailed results of phenotype comparisons are provided in Table S9. No other statistically significant differences in phenotype frequencies were detected. Overall, genetic differentiation among WB subpopulations was low across the analyzed pharmacogenes, indicating a high degree of similarity. Accordingly, for subsequent analyses, these subpopulations were considered independently and as a single WB population.

### 2.4. Comparison of Haplotype Frequencies with European Populations

Among the 77 analyzed pharmacogenes, population-specific allele frequencies for European populations were obtained using three approaches. For 48 genes (including *ABCB1*, *CFTR*, multiple *CYP* genes, *DPYD*, *GSTM1*, *GSTP1*, *TPMT*, *VKORC1*, and others), allele frequencies were derived from previously published pharmacogenetic analyses of structural variation in the 1000 Genomes dataset [11]. For 29 genes (*ABCG2*, *ADRA2*, *ADRB2*, *ANKK1*, *APOE*, *ATM*, *BCHE*, *BDNF*, *DBH*, *DRD2*, *F2*, *GRIK1*, *GRIK4*, *GRIN2B*, *HTR1A*, *HTR2A*, *IFNL3*, *IFNL4*, *ITGB3*, *ITPA*, *MTHFR*, *OPRK1*, *OPRM1*, *RARG*, *RYR1*, *SLC6A4*, *SLC28A3*, *SLC47A2*, and *UGT1A6*), allele frequencies were obtained from the 1000 Genomes Project data directly. For the four remaining genes (*COMT*, *CYP4F2*, *NAT1*, and *NAT2*), allele frequencies were estimated using the LDpair tool. For MT-*RNR1* and *GSTT1*, population-specific allele frequencies for European populations could not be reliably obtained. A Bonferroni correction for multiple comparisons was applied (0.05/77; *p* < 0.0006). Significant differences in haplotype frequencies were identified in 14 of the 77 pharmacogenes analyzed. Of these, 11 genes showed significant differences in frequency between the WB population and either the entire European population or certain European subpopulations (Figure 3).

Among clinically relevant haplotypes and variants (CPIC Levels A–B or ClinPGX Levels 1–2), four showed higher frequencies in the WB population or subpopulations compared to other European populations. These included *RYR1* (rs35364374 and rs55876273), which was more frequent in the WB population compared to the British population and Finnish populations, and *VKORC1* (rs9923231), which was more frequent compared to overall European and Finnish populations. In addition, CYP2C19*2B, was more frequent in the MNE subpopulation compared to European populations and CYP2B6*6, was more frequent in the B&H subpopulation compared to the Finnish population. A complete list of variants from all pairwise comparisons is presented in Table S10.

### 2.5. Comparison of Phenotype Frequencies with European Populations

In addition to haplotype comparisons, phenotype frequencies were evaluated for genes with established genotype–phenotype relationships according to CPIC guidelines (*n* = 16) and compared between the WB population and its subpopulations and European population and its subpopulations. Nine genes (*CYP2B6*, *CYP2C19*, *CYP2C9*, *CYP2D6*, *CYP3A5*, *DPYD*, *NUDT15*, *TPMT*, and *UGT1A1*) were used to assign metabolizer phenotypes (poor, intermediate, normal, rapid, and ultrarapid metabolizers). *CFTR* and *F5* were associated with therapeutic response phenotypes (ivacaftor, eltrombopag, respectively), while *ABCG2* and *SLCO1B1* were used to assign transporter function phenotypes (decreased, normal, or increased function). *CACNA1S* and *RYR1* were evaluated for variants associated with susceptibility to malignant hyperthermia. A Bonferroni correction for multiple comparisons was applied (0.05/16) and statistical significance was defined as *p* < 0.0031. Only one statistically significant difference was observed: the B&H subpopulation exhibited a higher frequency of the *CYP2B6* poor metabolizer phenotype compared to both the Finnish (*p* = 0.000008) and overall European (*p* = 0.0013) populations. All pairwise comparisons are presented in Table S11.

### 2.6. Functional Impacts of Novel Variants

To utilize the full extent of WGS data, after haplotype calling with pharmacogenomics tools, we also analyzed variants not classified with standard pharmacogenetic notation. We identified a total of 22,638 unique single-nucleotide variants across 33 clinically relevant pharmacogenes (CPIC Levels A–B or ClinPGX Levels 1–2). Of these, 472 variants were located within coding regions. Based on VEP predicted functional consequences, 229 variants were classified as having low impact, 229 as moderate impact, and 14 as high impact on protein function (Figure 4).

Among the moderate and high-impact variants, 95 were present in ClinPGx as standalone variants or as a part of star alleles, 147 were reported in ClinVar, and 237 were present in dbSNP, with six variants not previously reported in any of these databases. To evaluate the potential clinical relevance of the 148 moderate and high-impact variants not included in established star allele nomenclature and clinical databases (ClinPGx and ClinVar), variants were further filtered based on predicted functional consequences. MAFs were calculated for the WB population and compared with non-Finnish European and global populations using gnomAD. Four variants were classified as high impact by VEP, suggesting substantial disruption of protein function. These included three frameshift variants: two novel variants in *CYP2D6* (p.Arg269ProfsTer5 and p.Leu395HisfsTer11) and one in *NUDT15* (p.Gln6SerfsTer15), all predicted to introduce premature stop codons and likely result in loss of function via truncated proteins or nonsense-mediated decay. In addition, a splice acceptor variant in *CYP4F2* (c.344-1G>A) was identified and supported by SpliceAI (acceptor loss score = 0.93) and a high CADD Phred score (23), indicating a likely deleterious effect. Missense variants with strong evidence of pathogenicity were also observed. Several variants showed concordant predictions across multiple algorithms (PolyPhen-2, AlphaMissense, and CADD). Comparison with population databases showed that most identified variants were rare or absent in gnomAD (MAF < 0.01%). However, several variants exhibited relatively higher frequencies in the WB cohort, particularly in the *GRK4* gene, where the c.1370T>C (p.Leu457Pro) variant occurred with a frequency of 2.2%, similar to its ~2% frequency in the European population, while the variant c.182C>T (p.Pro61Leu) occurred with a frequency of 1%, which was almost three times higher than in the European population. Both variants were predicted to be pathogenic across all three prediction tools. In total, 22 variants exceeded a CADD Phred score of 20, placing them within the top 1% of predicted deleterious variants [16], these are summarized in Table 1. A complete list of all detected variants is provided in Table S12.

## 3. Discussion

PGx is an important precision medicine tool, as population- and individual-level genetic variability can influence drug response and toxicity, thereby informing more effective and safer treatment strategies [17]. This is the first comprehensive population PGx study of the WB region, encompassing five subpopulations from B&H, Croatia (CRO), MNE, NMC, and SRB. Our analyses positioned WB populations within the broader European genetic landscape, while identifying a number of distinctions with relevance for drug prescribing.

As expected, PCA across whole genome variants demonstrated a high degree of similarity between the entire WB population and the European population, while clear divergence was observed from the African, American and South and East Asian populations. Similar clustering was observed when analyzing only pharmacogenetic variants, showing that the pattern of pharmacogenetic variability closely follows populational differences. Within European subpopulations, the WB population showed particularly close genetic proximity to the Italian and Iberian subpopulations suggesting a shared genetic background. Looking more closely at the five WB subpopulations, we found they exhibit close similarity, which suggests only minor genetic differentiation within the region, and supports the presence of substantial common ancestry among Western Balkan populations. These results echo previous populational genetics studies performed on the WB population, where it was shown that the genetic profile of WB subpopulations resembles that of their geographical neighbors [18].

The annotation tools showed generally high concordance, with most genes demonstrating complete or near-complete agreement, supporting the reliability of WGS-based pharmacogenomic profiling when consensus approaches are applied [19,20]. However, discordance was observed for a subset of genes, reflecting structural variation, phasing ambiguity or inconsistent variant interpretation between tools. Application of consensus approaches improved concordance across several genes, particularly those affected by allele definition discrepancies and structural variation, although challenges remained for complex loci such as *CYP2D6*.

A significant difference in the frequency of the GSTP1*B allele was observed between the B&H and NMC subpopulations, with a higher frequency detected in B&H. No other significant differences in star allele frequencies were identified among the analyzed WB subpopulations, suggesting a relatively homogeneous pharmacogenomic landscape across the region. Although *GSTP1* is involved in the response to several chemotherapeutic agents, including cyclophosphamide, epirubicin, fluorouracil, and oxaliplatin, the current level of pharmacogenomic evidence for this gene remains limited. Therefore, at present, *GSTP1* is not routinely recommended for inclusion in clinical pharmacogenetic reports.

A higher frequency of *CYP2B6* poor metabolizer phenotypes, characterized by reduced enzymatic activity, was observed in the B&H subpopulation compared to the other analyzed WB groups. This trend was mainly driven by the increased frequency of the CYP2B6*6 allele, 36% in the B&H subpopulation, compared to 23.2% in the combined WB cohort, although the observed differences in our study did not reach statistical significance. Similar frequencies have previously been reported in CRO (24.5%) [19] and SRB (27.5%) [15] populations. Variants in *CYP2B6* are known to influence the metabolism and therapeutic response to several clinically important drugs, including sertraline, methadone, efavirenz, bupropion, and nevirapine. Importantly, the CYP2B6*6 variant has been associated with lower cyclophosphamide clearance, raising the possibility that its higher frequency in WB populations could contribute to interindividual differences in cyclophosphamide exposure and, consequently, treatment response and toxicity [20,21]. Moreover, *CYP2B6* activity has also been implicated in the response to certain antidepressants and anxiolytics [22]. For individuals predicted to be poor metabolizers, careful dose titration may be required to optimize therapy and reduce the risk of adverse drug reactions [23]. Collectively, these findings emphasize the importance of considering population-specific pharmacogenomic variability when interpreting drug response profiles within WB populations.

Significant differences in star allele frequencies between the WB and other European populations were identified in 15 pharmacogenes. Most of these differences involved genes with limited pharmacogenomic evidence. However, four genes (*CYP2B6*, *CYP2C19*, *RYR1*, and *VKORC1*) are classified as CPIC Level A or B and ClinPGx Level 1 or 2, highlighting their potential clinical relevance. Among these, the WB population showed higher frequencies of clinically relevant variants, including rs35364374 in *RYR1* and rs9923231 in *VKORC1*, while rs55876273 in *RYR1* was less frequent than in other European populations. In addition, *CYP2B6* *6 was more frequent in the B&H subpopulation and *CYP2C19* *2B in the MNE subpopulation. CYP2B6*2B is a suballele of *2 rather than a distinct functional variant, and both are classified as no-function alleles, so this substitution has no consequence for phenotype assignment or for genotype-guided prescribing. Our findings for *VKORC1* are consistent with several previous studies from the WB region, which also [15,24,25,26,27] reported elevated frequencies of rs9923231. Given the role of *VKORC1* in warfarin sensitivity and dose requirements, genotyping rs9923231 before initiating coumarin anticoagulants may be of greater benefit in WB patients than in other Europeans, reducing the risk of over-anticoagulation and bleeding. This is particularly relevant given the narrow therapeutic index of warfarin and the potential consequences of inappropriate dosing. For *RYR1*, rs35364374 was more frequent in the WB cohort than in the FIN population, whereas rs55876273 was less frequent than in other European populations, consistent with previous findings in the SRB population [15]. Although *RYR1* is a major gene associated with malignant hyperthermia susceptibility, only specific *RYR1* and *CACNA1S* missense variants are currently established as clinically actionable [28]. Both variants identified here are of uncertain significance, and functional characterization will be required before these frequency differences can inform malignant hyperthermia risk assessment in WB patients. The higher frequencies of *CYP2B6* *6 in B&H and *CYP2C19* *2B in MNE were not consistently reported in previous studies [15,19,29] and may partly reflect population-specific variation or differences in sample size. The increased frequency of *CYP2B6* *6 in B&H is of particular clinical interest, as CPIC guidelines support genotype-guided efavirenz and sertraline dosing, including careful dose consideration in poor metabolizers and in certain combinations of *CYP2B6* [23]. Testing for *6, a decreased-function allele, would therefore be of particular value in B&H patients, in whom carriers are at risk of elevated efavirenz and sertraline exposure at standard doses.

The comprehensive nature of whole genome data also enabled the discovery of rare and novel variants in pharmacogenes, which are often missed by star allele calling tools and pharmacovariant profiling. We identified 148 variants with functional impact within clinically relevant pharmacogenes that have previously not been reported in either pharmacogenetic or clinical variant databases. This makes up close to one third of all detected coding sequence variants within pharmacogenes, most of which were rare, occurring only in a single or a few individuals. This is in accordance with what was shown in studies on public databases such as ExAc and 1kGP, which state that most functional variants in pharmacogenes are observed with frequencies below 0.1% and thus cannot be observed in clinical trials. This highlights the potential for a holistic approach that takes into account all functional variants, as even though they are individually rare they make up a significant portion of pharmacogenetic variation across the population. We also observed two more common unreported missense variants in the *GRK4* gene, one of which is consistent with other European populations, while the other was more frequent in our WB cohort, highlighting potential population specificity. CPIC guidelines for this gene state that there is a lack of studies related to individual variants and their associations with beta-blocker response [30]. It should, however, be noted that protein impact scores such as the ones used in this study to score missense variants might not always be the ideal tool for understanding the potential impact of variants on pharmacogene function, as they boil down to deleteriousness scores, and cannot be used to predict increases in metabolic activity that some pharmacogenetic variants are known to cause.

This study has several limitations that should be acknowledged. First, it was conducted on a relatively small sample, which may limit the detection of the rare variants and limit the broader applicability of the findings. However, it is important to emphasize that this work represents the first population-scale pharmacogenomic investigation in the WB region, and to our knowledge, the first analysis of WGS data in this population from a pharmacogenetic perspective. To mitigate the limitation related to sample size, we compared our cohort with the 1000 Genomes dataset, selecting European subpopulations with matched sample sizes, thereby enabling more balanced and comparable analyses. Although the relatively small sample size reduces statistical power, statistically significant population differences can still be detected when allele-frequency differences are sufficiently pronounced [27]. Additionally, highly polymorphic *HLA* genes were not included in this study, which represents another limitation given their known relevance in pharmacogenetics, particularly in drug hypersensitivity reactions. Despite these limitations, this study has several important strengths. We comprehensively analyzed 88 pharmacogenes, encompassing all genes that could be reliably annotated using the applied tools, providing a broad overview of pharmacogenomic variability in this population. Furthermore, we employed multiple complementary analytical approaches, including population comparisons, principal component analysis, and the identification of novel variants, which together enhance the robustness and depth of our findings. Future studies with larger cohorts and inclusion of complex genomic regions such as *HLA* are warranted to provide a more comprehensive understanding of pharmacogenomic variability in this population.

## 4. Materials and Methods

### 4.1. Subjects

For the population analysis of the WB region, we used a genomic repository generated within the PharmGenHUB project. The repository included DNA extracted from blood samples from in total 250 healthy individuals from each of the five countries: B&H (University Clinical Centre of the Republic of Srpska, Banja Luka), CRO (Department of Pediatrics, Clinical Hospital Centre Rijeka, Rijeka), MNE (Center for Medical Genetics and Immunology, Clinical Center of Montenegro, Podgorica), NMC (University Clinic for Pediatrics, Faculty of Medicine, Skopje), and SRB (Institute of Molecular Genetics and Genetic Engineering, University of Belgrade, Belgrade) (Supplementary Table S1). Although samples were collected through participating institutions across the five countries, all DNA processing, WGS, and subsequent genomic data generation were performed at the Institute of Molecular Genetics and Genetic Engineering (IMGGE), University of Belgrade, Serbia, using the same laboratory workflow and sequencing platform. Participants were recruited from the adult population, aiming at balanced gender representation and the inclusion of healthy, unrelated individuals. Written informed consents were obtained from all participants for the use of their genomic data in pharmacogenomics research. The study was approved by the Ethical Committees of the Institute of Molecular Genetics and Genetic Engineering, University of Belgrade (No. O-EO-046/2023 from 9 March 2023), the Clinical Hospital Centre Rijeka (No. 2170-29-02/1-23-4 from 28 July 2023), the University Clinical Centre of the Republic of Srpska (No. 01-19-157-2/23 from 26 April 2023), the Clinical Center of Montenegro (No. 1832/4 from 10 November 2023), and the Faculty of Medicine, Skopje (No. 03-1704/1 from 5 September 2023).

Publicly available genotyping data from the 1000 Genomes Project were used for comparative analyses. Specifically, European populations (*n* = 503) were included: Utah residents with Northern and Western European ancestry (CEU), British in England and Scotland (GBR), Finnish in Finland (FIN) Iberian populations in Spain (IBS), and Toscani in Italy (TSI). Pharmacogenomic data were obtained from the 1000 Genomes Project Phase 3 release “http://www.1000genomes.org (accessed on 17 November 2025)” and from previously published work [11] on pharmacogenetic analysis of structural variation in the 1000 Genomes dataset. In cases of discrepancies between these two data sources, variant calls were manually verified by inspecting Variant Call Format (VCF) files from the 1000 Genomes dataset, and genotypes were independently reassessed.

### 4.2. Whole Genome Sequencing and Data Processing

All samples underwent WGS using the Illumina DNA PCR-Free Prep kit (Illumina, San Diego, CA, USA) and were sequenced using an Illumina sequencing platform. The average sequencing depth across the whole genome was 40X, with an average of 75% of the genome calling regions being covered with 30X depth or higher. All samples had over 90% of genomic regions covered with at least 10X depth.

Raw WGS data were processed using an in-house variant calling pipeline following GATK Best Practices [31]. In short, the pipeline included quality control of FASTQ reads with the FASTP tool, alignment to the hg38 reference genome with BWA-MEM to generate raw BAM files, and alignment processing in the form of marking duplicates (GATK MarkDuplicates) and base quality score recalibration (GATK BaseRecalibrator and ApplyBQSR). Generated analysis-ready BAM files were used for per sample variant calling with HaplotypeCaller producing GVCF files, which were then combined into a GenomicsDB and cohort variant calling was performed with GenotypeGVCFs. This produced a cohort-level multi-sample VCF file which was filtered using VQSR, to generate high-quality variant calls.

Since VCF files contain only information about variable sites, all relevant PGx positions important for star allele annotation have been examined for their quality and depth in BAM files, ensuring that PGx annotation tools that operated only with VCF files (such as PharmCAT) made correct calls by assuming reference alleles.

### 4.3. Star Allele Identification

Star alleles were inferred from WGS data across 88 pharmacogenes, listed in Table S4. Star allele calling and pharmacogenomic annotation were performed using four tools: Aldy4 [32,33], PharmCAT [34], PyPGx [35], and Stargazer [36,37], with the latter applied exclusively for *CYP2D6* genotyping (Supplementary Table S2). Aldy4 (v.4.8.2.) was used for genotyping 38 highly polymorphic pharmacogenes with complex structural variation, enabling allele decomposition and copy number-aware star allele assignment, using the WGS-specific configuration as described by Hari et al. [32,33]. This is achieved by reconstructing haplotypes directly from BAM files by integrating information from single nucleotide variation (SNVs), read-backed phasing, and coverage profiles, and matching them to curated allele definition databases. PharmCAT (v.3.0.1.) was applied to the processed multi-sample VCF files, to infer haplotypes and assign star allele nomenclature for 18 clinically relevant pharmacogenes based on definitions from the PharmVar Consortium and CPIC guidelines. PyPGx (v.0.25.0.) was used to call star alleles across 88 pharmacogenes by integrating SNV and copy number variation (CNV) profiles to enable comprehensive haplotype and diplotype assignment in pharmacogenes. Similarly to Aldy4, SNVs were phased using Beagle and combined into haplotypes per individual, while CNVs are estimated from read-depth data using intra-sample normalization against the *VDR* gene as the control region. Stargazer (v2.0) was used exclusively for *CYP2D6* genotyping to provide further support in the detection of complex structural rearrangements and copy-number variation at this locus.

All four tools translate variant combinations into diplotypes and, where applicable, assign predicted phenotypes according to PharmVar and CPIC guidelines. Variants not matching defined allele patterns or located in poorly covered regions were reported as NA or Indeterminate according to tool-specific criteria. Concordance between tools was assessed, and discrepancies were resolved by manual inspection of the underlying BAM files where necessary. All analyses were performed using consistent tool versions and allele definition databases to ensure reproducibility. The set of analyzed genes was selected based on clinical relevance (CPIC, FDA), curated allele definition resources (PharmVar, ClinPGx), and overlap with established pharmacogenomic panels [35,38].

### 4.4. Population Structure Analysis

Population structure analysis was conducted using principal component analysis (PCA) across the studied cohorts. Genotype data from the five WB populations and from all populations in the 1000 Genomes Project dataset were merged and pruned using PLINK software to remove variants in strong linkage disequilibrium (LD) and to perform quality control of the analyzed cohort on the whole genome level. Both the regional and the worldwide genetic structure were evaluated by conducting a PCA separately for the European populations and the global populations. Further PCAs were restricted to the variants located in the coding regions of pharmacogenes to estimate the pharmacogenetic structure of the WB populations in comparison to other European populations.

### 4.5. Novel Variant Detection

Filtered multi-sample VCF files were analyzed using a variant annotation framework. Rare and previously unreported variants, as well as variants of unknown functional significance with potential relevance to pharmacologic response, were selected for further analysis. Variant annotation was performed using Ensembl Variant Effect Predictor (VEP) [39], incorporating variant classification, population frequency, reporting in clinical and pharmacogenomic databases, and functional effect prediction algorithms. Population allele frequencies were obtained from the Genome Aggregation Database (gnomAD) [40], while known clinical and pharmacogenomic associations were retrieved from ClinVar and ClinPGx, respectively. Variants that have not been previously reported in either ClinVar or ClinPGx were considered novel for the purpose of pharmacogenetic potential. Functional impact predictions for high and moderate impact variants were derived from multiple in silico tools, including PolyPhen-2 [41], AlphaMissense [42], and CADD [16]. SpliceAI [43] was used to predict the impact of variants on splicing. These variants were then ranked based on VEP impact and predicted functional effect scores to identify candidates with a high likelihood of deleterious effects.

### 4.6. Statistical and Population Genetic Analysis

All statistical analyses were performed in R (version 4.4.0). Initial genotype data quality was assessed by testing for deviations from HWE, including multiallelic loci. Due to the highly polymorphic nature of several pharmacogenes, HWE was evaluated using a Monte Carlo permutation approach (B = 1000), implemented via the hw.test function in the *pegas* package, which is appropriate for multiallelic data where asymptotic tests are not applicable. The significance threshold was adjusted for multiple testing by dividing 0.05/77 by the number of pharmacogenes with at least two detected alleles, resulting in an adjusted *p*-value threshold of <0.0006. Pharmacogenes in which only reference alleles were detected (*ACYP2*, *CACNA1S*, *CYP2R1*, *CYP17A1*, and *CYP26A1*) were excluded from further comparative analyses. HWE was assessed separately for each of the five Western Balkan subpopulations and for the combined WB cohort (Table S13). To ensure a consistent set of pharmacogenes for cross-population comparisons, genes showing significant deviation from HWE in at least one individual WB subpopulation were excluded from subsequent comparative analyses. Based on this criterion, *CYP2E1*, *G6PD*, *GSTT1*, *IFNL3*, and *SULT1A1* were excluded. The principal component analysis did not indicate substantial genetic differentiation among the five WB subpopulations, suggesting that pronounced population stratification was unlikely to account for these deviations. Due to a significant deviation from HWE these 5 genes were excluded from the final comparative analysis. A final list of 78 genes that come into further analysis for WB subpopulation comparison are in Table S4.

Population pharmacogenomic analyses compared star allele frequencies in the WB population with those observed in European populations from the 1000 Genomes Project, described above [11]. Frequency comparisons were performed using Fisher’s exact test. For genes where haplotype frequencies could not be directly obtained from published 1000 Genomes data (*COMT*, *NAT1*, *NAT2*), haplotype frequencies were estimated using the LDpair tool [44], which derives linkage disequilibrium–based frequencies from 1000 Genomes data. Predicted phenotypes for actionable pharmacogenes were obtained from the star allele calling tools described above, and for the genes not supported by these tools (*NAT1*, *NAT2*, *MT-RNR1*), phenotype information was retrieved from publicly available pharmacogenomic resources “https://nat.mbg.duth.gr/Homepage.htm (accessed on 1 December 2025)” and CPIC guidelines. Phenotype frequencies were also compared using Fisher’s exact test. In both cases, Bonferroni correction was applied to adjust for multiple comparisons by dividing the significance threshold (0.05) by the number of pharmacogenes tested, thereby reducing the likelihood of false-positive results.

## 5. Conclusions

This study provides the first population-scale, WGS-based characterization of pharmacogenomic variability in the Western Balkans. While the WB population aligns closely with other European populations overall, we identified distinctions of potential clinical importance. Among the genes that differed, the most clinically relevant were *CYP2B6*, *CYP2C19*, *RYR1*, and *VKORC1*, all supported by highest pharmacogenomic evidence and actionable prescribing guidance. Beyond established star alleles, whole genome data revealed a substantial burden of rare and previously unreported variants with potential functional impact, underscoring the value of a comprehensive, variant-level approach to pharmacogenomic profiling. These findings establish a foundation for integrating pharmacogenomics into clinical practice in the WB and highlight the importance of including underrepresented populations in pharmacogenomic research.

## Figures and Tables

**Figure 1:**
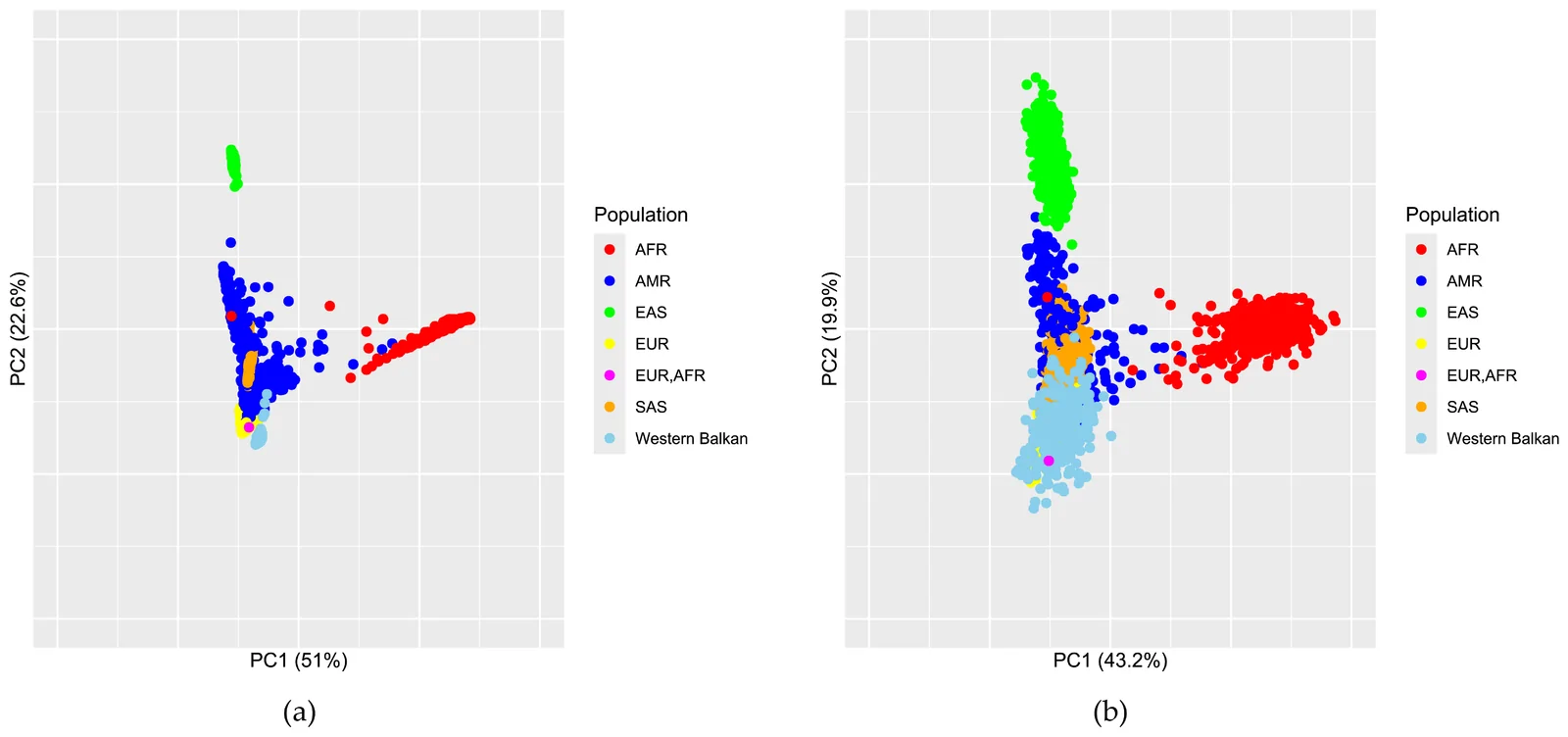
Principal component analysis (PCA) plot of the Western Balkan subpopulation and 1000 genome project populations originating from Sub-Saharan Africa (AFR), America (AMR), East Asian (EAS), Europe (EUR), European/African (EUR/AFR), South Asian (SAS) populations. (**a**) PCA on whole-genome data; (**b**) PCA on subset regions of the pharmacogenes.

**Figure 2:**
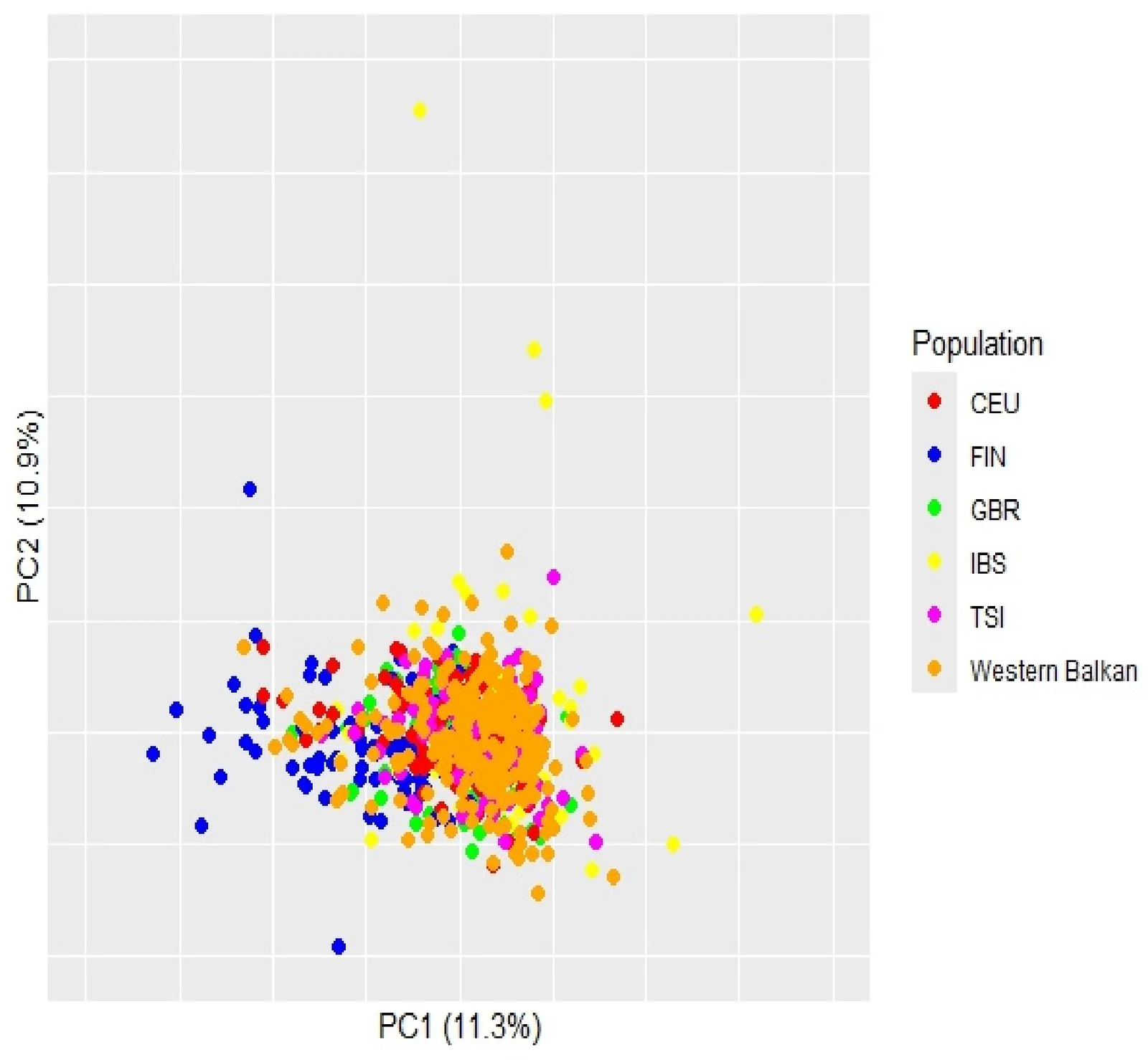
Principal component analysis on subset regions of the pharmacogenes of the Western Balkan subpopulation in comparison with 1000 Genomes Project reference populations, including Utah residents with Northern and Western European ancestry (CEU), British in England and Scotland (GBR), Finnish (FIN), Iberian populations in Spain (IBS), Toscani in Italy (TSI).

**Figure 3:**
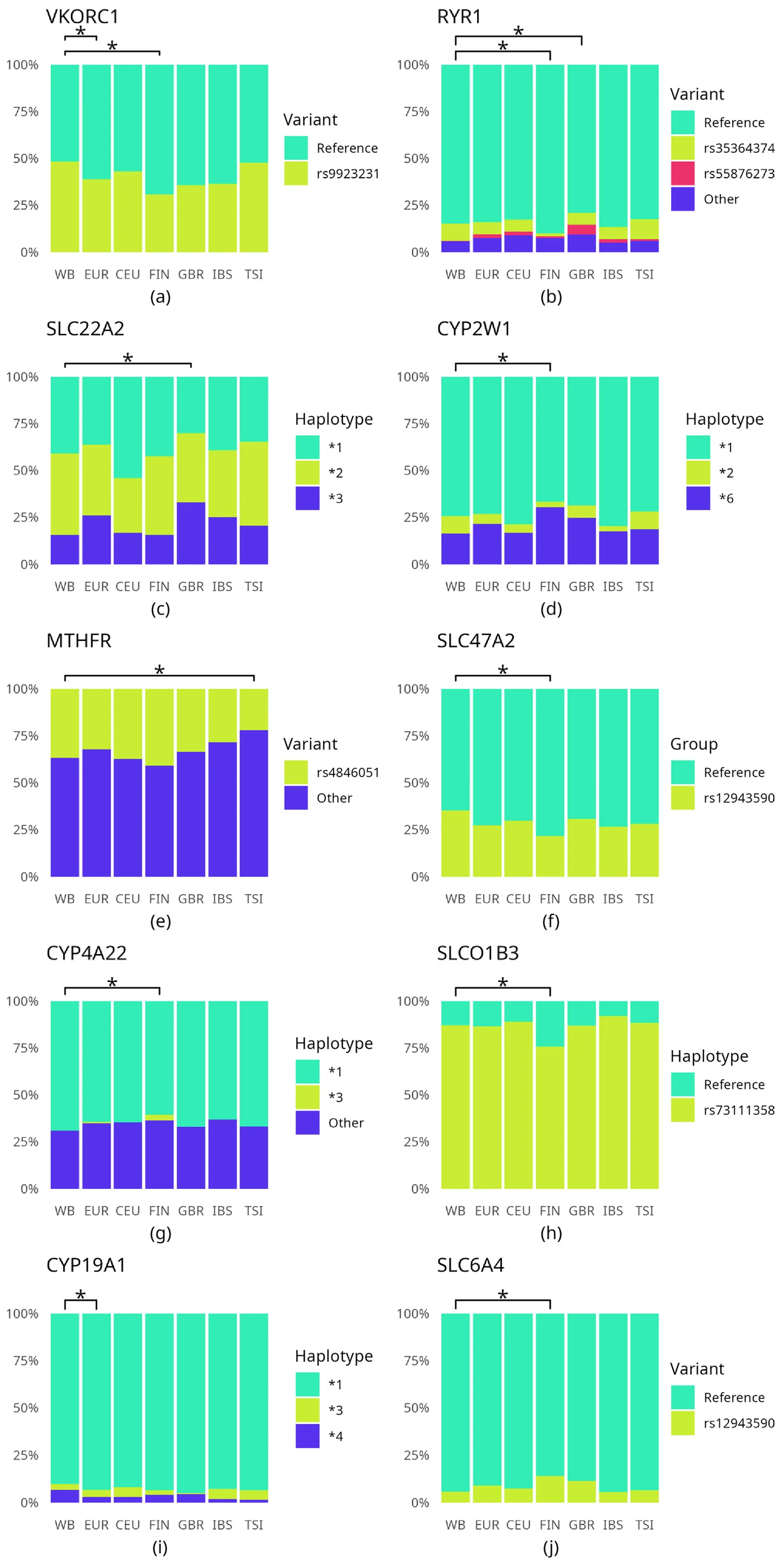
Star alleles and variants in pharmacogenes showing significantly different frequencies in the Western Balkan (WB) population compared to European population and its subpopulations. CEU—Utah residents with Northern and Western European ancestry; GBR—British in England and Scotland; EUR—European populations; FIN—Finnish in Finland; IBS—Iberian populations in Spain; TSI—Toscani in Italy. (**a**) *VKORC1* variant rs9923231; (**b**) *RYR1* variants rs35364374 and rs55876273; (**c**) *SLC22A2* haplotype *3; (**d**) *CYP2W1* *6; (**e**) *MTHFR* variant rs4846051; (**f**) *SLC4A2* variant rs12943590; (**g**) *CYP4A22* haplotype *3; (**h**) *SLCO1B3* variant rs73111358; (**i**) *CYP19A1* haplotype *4; (**j**) *SLC6A4* variant rs12943590. *—significant threshold *p* < 0.0006.

**Figure 4:**
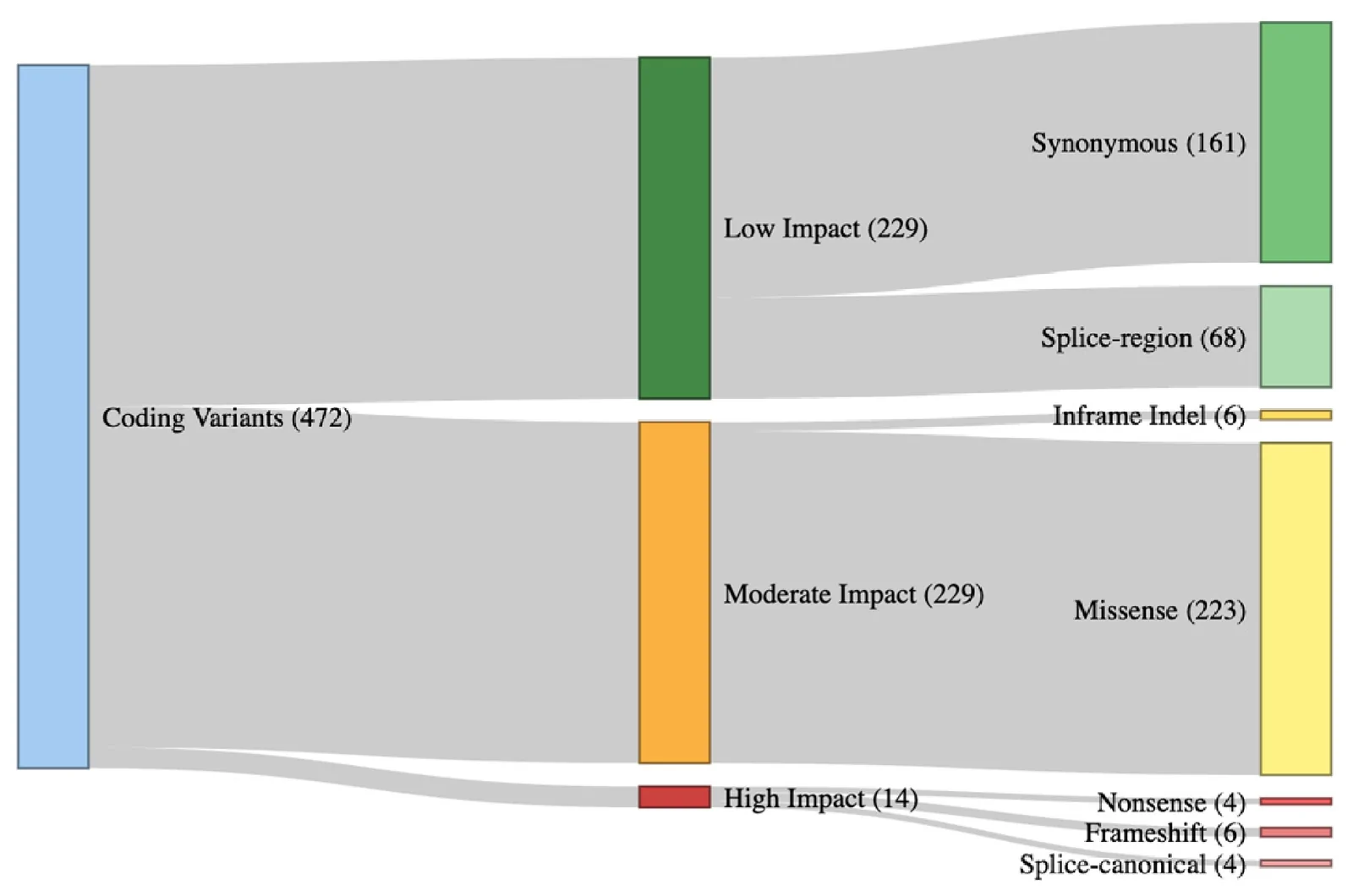
Sankey diagram illustrating the distribution of coding variants (*n* = 472) based on their predicted impact on protein function (low, moderate, and high), with further classification into specific variant consequences, including synonymous, splice-region, inframe indels, missense, nonsense, frameshift, and splice-cannonical variants.

**Table 1:** Predicted deleterious variants in genes with CPIC Levels A–B or ClinPGX Levels 1–2, prioritized by high CADD scores and functional consequence.

Position	rs ID	CPIC Gene	HGVS	VEP Impact	Consequence	PolyPhen	Alpha Missense	CADD Phred	SpliceAI	Predicted Pathogenic	WB Allele Count	WB AF	gnomADg AF (NFE)	gnomADg AF (Global)
chr13:48037761 delA	rs1566105829	NUDT15	NM_018283.4:c.15del (p.Gln6SerfsTer15)	High	Frameshift	-	-	NA			2	0.4%	0.000%	0.000%
chr22:42128212 dupG	rs72549352	CYP2D6	NM_000106.6:c.805dup (p.Arg269ProfsTer5)	High	Frameshift	-	-	29.7			1	0.2%	0.016%	0.019%
chr22:42126981 delGA	rs757396767	CYP2D6	NM_000106.6:c.1184_118del (p.Leu395HisfsTer11)	High	Frameshift	-	-	27.5			1	0.2%	0.019%	0.009%
chr19:15892583 C/T	rs763450661	CYP4F2	NM_001082.5:c.344-1G>A	High	Splice acceptor	-	-	23	Acceptor Loss (0.93)		1	0.2%	0.003%	0.001%
chr2:233767933 C/T	-	UGT1A1	NM_000463.3:c.1081C>T (p.Leu361Phe)	Moderate	Missense	probably damaging (1)	likely pathogenic (0.9378)	29.8		**3 of 3**	1	0.2%	0.000%	0.000%
chr4:3037482 G/C	rs767668126	GRK4	NM_182982.3:c.1516G>C (p.Gly506Arg)	Moderate	Missense	probably damaging (0.996)	likely pathogenic (0.7585)	28.1		**3 of 3**	1	0.2%	0.000%	0.000%
chr19:15885975 C/T	rs375460865	CYP4F2	NM_001082.5:c.1064G>A (p.Arg355Gln)	Moderate	Missense	probably damaging (0.984)	likely benign (0.1269)	27.7		2 of 3	1	0.2%	0.004%	0.011%
chr6:154091079 G/T	rs201750403	OPRM1	NM_000914.5:c.771G>T (p.Met257Ile)	Moderate	Missense	probably damaging (1)	likely pathogenic (0.9922)	27.4		**3 of 3**	1	0.2%	0.003%	0.006%
chr4:3035486 T/C	rs34022679	GRK4	NM_182982.3:c.1370T>C (p.Leu457Pro)	Moderate	Missense	probably damaging (1)	likely pathogenic (0.9524)	27.2		**3 of 3**	11	2.2%	1.939%	1.450%
chr13:46835562 C/T	rs914903401	HTR2A	NM_000621.5:c.691G>A (p.Asp231Asn)	Moderate	Missense	probably damaging (0.956)	likely benign (0.2114)	27		2 of 3	1	0.2%	0.000%	0.000%
chr10:119452735 A/G	-	GRK5	NM_005308.3:c.1469A>G (p.Asn490Ser)	Moderate	Missense	probably damaging (0.979)	likely benign (0.0809)	26.4		2 of 3	1	0.2%	0.000%	0.000%
chr7:117627549 T/C	rs754847820	CFTR	NM_000492.4:c.3496T>C (p.Phe1166Leu)	Moderate	Missense	possibly damaging (0.517)	likely pathogenic (0.9287)	26.1		2 of 3	1	0.2%	0.000%	0.001%
chr4:2988760 C/T	rs140178959	GRK4	NM_182982.3:c.182C>T (p.Pro61Leu)	Moderate	Missense	probably damaging (1)	likely pathogenic (0.5962)	25.7		**3 of 3**	5	1.0%	0.372%	0.264%
chr13:48037822 C/G	rs561923692	NUDT15	NM_018283.4:c.76C>G (p.Pro26Ala)	Moderate	Missense	probably damaging (0.916)	likely benign (0.1753)	25.2		2 of 3	1	0.2%	0.004%	0.002%
chr19:15879413 G/A	rs142113670	CYP4F2	NM_001082.5:c.1330C>T (p.Arg444Cys)	Moderate	Missense	probably damaging (0.923)	likely benign (0.2893)	23.8		2 of 3	2	0.4%	0.022%	0.016%
chr19:39243685 G/A	rs77379751	IFNL3	NM_172139.4:c.538C>T (p.Arg180Cys)	Moderate	Missense	probably damaging (1)	likely benign (0.3048)	23.8		2 of 3	1	0.2%	0.071%	0.438%
chr7:99672587 T/C	-	CYP3A5	NM_000777.5:c.311A>G (p.Asn104Ser)	Moderate	Missense	probably damaging (0.994)	likely benign (0.2508)	23.8		2 of 3	1	0.2%	0.000%	0.000%
chr2:233767876 C/A	rs773195449	UGT1A1	NM_000463.3:c.1024C>A (p.Pro342Thr)	Moderate	Missense	possibly damaging (0.888)	likely pathogenic (0.9033)	23.6		2 of 3	1	0.2%	0.004%	0.002%
chr10:94988916 A/G	rs748356078	CYP2C9	NM_000771.4:c.1361A>G (p.Gln454Arg)	Moderate	Missense	probably damaging (0.997)	likely benign (0.2594)	22.3		2 of 3	1	0.2%	0.000%	0.001%
chr19:39244114 G/A	rs145428712	IFNL3	NM_172139.4:c.302C>T (p.Thr101Met)	Moderate	Missense	probably damaging (0.96)	likely benign (0.1815)	22		2 of 3	5	1.0%	0.184%	0.130%
chr10:95067319 G/A	rs377386087	CYP2C8	NM_000770.3:c.370C>T (p.Arg124Trp)	Moderate	Missense	possibly damaging (0.9)	likely pathogenic (0.7531)	21.2		2 of 3	2	0.4%	0.006%	0.005%

Note: CPIC—Clinical Pharmacogenetics Implementation Consortium; HGVS—Human Genome Variation Society; VEP—Variant Effect Predictor; CADD—Combined Annotation-Dependent Depletion Score; WB—Western Balkan; gnomADg—Genom Aggregation Database Genomes, AF—allele frequency.

## Data Availability

The original contributions presented in the study are included in the article/Supplementary Material, further inquiries can be directed to the corresponding author.

## Supplementary Materials

The following supporting information can be downloaded at: https://www.mdpi.com/article/10.3390/ph19101526/s1, Table S1: Subject characteristics (n = 250); Table S2: Comparison of genotype calls across pharmacogenomic annotation tools (Aldy, PyPGx, PharmCAT and Stargazer) and identified sources of discordance. Table S3: Genotyping. Table S4: Gene list. Table S5: Overview of genes. Table S6: WB haplotype frequency. Table S7: WB haplotype comparison. Table S8: WB phenotype frequency. Table S9: WB phenotype comparison. Table S10: EUR vs. WB haplotype comparison. Table S11: EUR vs. WB phenotype comparison. Table S12: CPIC novel pharmacovariants. Table S13: Hardy–Weinberg equilibrium.

## Abbreviations

The following abbreviations are used in this manuscript:

ADME	Absorption, Distribution, Metabolism, and Excretion
B&H	Bosnia and Herzegovina
BAM	Binary Alignment Map
CEU	Northern and Western European ancestry
ClinGen PGxWG	Clinical Genome Resource Pharmacogenomics Working Group
CNV	Copy number variation
CPIC	Clinical Pharmacogenetics Implementation Consortium
CRO	Croatia
EMA	European Medicines Agency
FDA	Food and Drug Administration
FIN	Finnish in Finland
GBR	British in England and Scotland
gnomAD	Genome Aggregation Database
HWE	Hardy–Weinberg equilibrium
IBS	Iberian populations in Spain
LD	Linkage disequilibrium
MNE	Montenegro
NGS	Next-Generation Sequencing
NMC	North Macedonia
PCA	Principal Component Analysis
PC	Principal components
PGx	Pharmacogenomics
QC	Quality control
SNV	Single nucleotide variation
SRB	Serbia
TSI	Toscani in Italy
VCF	Variant Call Format
VEP	Variant Effect Predictor
WGS	Whole-Genome Sequencing
WB	Western Balkan

## References

[1] Arbitrio M., Di Martino M.T., Scionti F., Barbieri V., Pensabene L., Tagliaferri P. (2018). Pharmacogenomic Profiling of ADME Gene Variants: Current Challenges and Validation Perspectives. High-Throughput.

[2] Relling M.V., Evans W.E. (2015). Pharmacogenomics in the Clinic. Nature.

[3] Barbarino J.M., Whirl-Carrillo M., Altman R.B., Klein T.E. (2018). PharmGKB: A Worldwide Resource for Pharmacogenomic Information. WIREs Syst. Biol. Med..

[4] Krebs K., Milani L. (2019). Translating Pharmacogenomics into Clinical Decisions: Do Not Let the Perfect Be the Enemy of the Good. Hum. Genome.

[5] Gong L., Klein C.J., Caudle K.E., Moyer A.M., Scott S.A., Whirl-Carrillo M., Klein T.E., Aminkeng F., Amr S., ClinGen Pharmacogenomics Working Group (PGxWG) (2025). Integrating Pharmacogenomics into the Broader Construct of Genomic Medicine: Efforts by the ClinGen Pharmacogenomics Working Group (PGxWG). Clin. Chem..

[6] Tibben B.M., Gaedigk A., Gong L., Sangkuhl K., Whirl-Carrillo M., Relling M.V., Donnelly R.S., Klein T.E., Caudle K.E. (2025). The Clinical Pharmacogenetics Implementation Consortium’s Consensus-Based Framework for Assigning Allele Function. Am. J. Hum. Genet..

[7] Van Der Lee M., Kriek M., Guchelaar H.-J., Swen J.J. (2020). Technologies for Pharmacogenomics: A Review. Genes.

[8] Ahmed S., Zhou Z., Zhou J., Chen S.-Q. (2016). Pharmacogenomics of Drug Metabolizing Enzymes and Transporters: Relevance to Precision Medicine. Genom. Proteom. Bioinform..

[9] Twesigomwe D., Wright G.E.B., Drögemöller B.I., Da Rocha J., Lombard Z., Hazelhurst S. (2020). A Systematic Comparison of Pharmacogene Star Allele Calling Bioinformatics Algorithms: A Focus on CYP2D6 Genotyping. npj Genom. Med..

[10] Halman A., Lunke S., Sadedin S., Moore C., Conyers R. (2024). Benchmarking Pharmacogenomics Genotyping Tools: Performance Analysis on Short-read Sequencing Samples and Depth-dependent Evaluation. Clin. Transl. Sci..

[11] Sherman C.A., Claw K.G., Lee S. (2024). Pharmacogenetic Analysis of Structural Variation in the 1000 Genomes Project Using Whole Genome Sequences. Sci. Rep..

[12] Lakiotaki K., Kanterakis A., Kartsaki E., Katsila T., Patrinos G.P., Potamias G. (2017). Exploring Public Genomics Data for Population Pharmacogenomics. PLoS ONE.

[13] McInnes G., Lavertu A., Sangkuhl K., Klein T.E., Whirl-Carrillo M., Altman R.B. (2021). Pharmacogenetics at Scale: An Analysis of the UK Biobank. Clin. Pharmacol. Ther..

[14] Petrović J., Pešić V., Lauschke V.M. (2020). Frequencies of Clinically Important CYP2C19 and CYP2D6 Alleles Are Graded across Europe. Eur. J. Hum. Genet..

[15] Jelovac M., Pavlovic D., Stankovic B., Kotur N., Ristivojevic B., Pavlovic S., Zukic B. (2025). Comprehensive Pharmacogenomics Profiling of the Serbian Population. Front. Pharmacol..

[16] Kircher M., Witten D.M., Jain P., O’Roak B.J., Cooper G.M., Shendure J. (2014). A General Framework for Estimating the Relative Pathogenicity of Human Genetic Variants. Nat. Genet..

[17] Jordan I.K., Sharma S., Mariño-Ramírez L. (2023). Population Pharmacogenomics for Health Equity. Genes.

[18] Kovacevic L., Tambets K., Ilumäe A.-M., Kushniarevich A., Yunusbayev B., Solnik A., Bego T., Primorac D., Skaro V., Leskovac A. (2014). Standing at the Gateway to Europe—The Genetic Structure of Western Balkan Populations Based on Autosomal and Haploid Markers. PLoS ONE.

[19] Matišić V., Brlek P., Bulić L., Molnar V., Dasović M., Primorac D. (2023). Population Pharmacogenomics in Croatia: Evaluating the PGx Allele Frequency and the Impact of Treatment Efficiency. Int. J. Mol. Sci..

[20] Veal G.J., Cole M., Chinnaswamy G., Sludden J., Jamieson D., Errington J., Malik G., Hill C.R., Chamberlain T., Boddy A.V. (2016). Cyclophosphamide Pharmacokinetics and Pharmacogenetics in Children with B-Cell Non-Hodgkin’s Lymphoma. Eur. J. Cancer.

[21] Mangó K., Fekete F., Kiss Á.F., Erdős R., Fekete J.T., Bűdi T., Bruckner E., Garami M., Micsik T., Monostory K. (2023). Association between CYP2B6 Genetic Variability and Cyclophosphamide Therapy in Pediatric Patients with Neuroblastoma. Sci. Rep..

[22] Radosavljevic M., Svob Strac D., Jancic J., Samardzic J. (2023). The Role of Pharmacogenetics in Personalizing the Antidepressant and Anxiolytic Therapy. Genes.

[23] Desta Z., Gammal R.S., Gong L., Whirl-Carrillo M., Gaur A.H., Sukasem C., Hockings J., Myers A., Swart M., Tyndale R.F. (2019). Clinical Pharmacogenetics Implementation Consortium (CPIC) Guideline for *CYP2B6* and Efavirenz-Containing Antiretroviral Therapy. Clin. Pharmacol. Ther..

[24] Kovac M.K., Maslac A.R., Rakicevic L.B., Radojkovic D.P. (2010). The c.-1639G>A Polymorphism of the VKORC1 Gene in Serbian Population: Retrospective Study of the Variability in Response to Oral Anticoagulant Therapy. Blood Coagul. Fibrinolysis.

[25] Celinšćak Ž., Zajc-Petranović M., Šetinc M., Stojanović Marković A., Peričić Salihović M., Zeljko H.M., Janićijević B., Smolej Narančić N., Škarić-Jurić T. (2022). Pharmacogenetic Distinction of the Croatian Population from the European Average. Croat. Med. J..

[26] Šupe S., Poljaković Z., Božina T., Ljevak J., Macolić Šarinić V., Božina N. (2015). Clinical Application of Genotype-Guided Dosing of Warfarin in Patients with Acute Stroke. Arch. Med. Res..

[27] Mizzi C., Dalabira E., Kumuthini J., Dzimiri N., Balogh I., Başak N., Böhm R., Borg J., Borgiani P., Bozina N. (2016). A European Spectrum of Pharmacogenomic Biomarkers: Implications for Clinical Pharmacogenomics. PLoS ONE.

[28] Silva H.C.A., Mendonça D.C., Souza B.W., Santos J.M., Souza L.S., Junior A.F.R., Vasconcelos F.T.G.R., Andrade P.V., Oliveira A.S.B., Vainzof M. (2025). Genetic Characteristics of Brazilian Patients with MH History. Genes.

[29] Perović S., Vujović S., Perović S., Šćepanović A. (2024). The Role of CYP2C19*2 Variant and Other Factors in Clopidogrel Resistance in Montenegrin ACS Patients. Genet. Appl..

[30] Duarte J.D., Thomas C.D., Lee C.R., Huddart R., Agundez J.A.G., Baye J.F., Gaedigk A., Klein T.E., Lanfear D.E., Monte A.A. (2024). Clinical Pharmacogenetics Implementation Consortium Guideline (CPIC) for *CYP2D6*, *ADRB1*, *ADRB2*, *ADRA2C*, *GRK4*, and *GRK5* Genotypes and Beta-Blocker Therapy. Clin. Pharmacol. Ther..

[31] Poplin R., Ruano-Rubio V., DePristo M.A., Fennell T.J., Carneiro M.O., Van Der Auwera G.A., Kling D.E., Gauthier L.D., Levy-Moonshine A., Roazen D. (2017). Scaling Accurate Genetic Variant Discovery to Tens of Thousands of Samples. bioRxiv.

[32] Hari A., Zhou Q., Gonzaludo N., Harting J., Scott S.A., Qin X., Scherer S., Sahinalp S.C., Numanagić I. (2023). An Efficient Genotyper and Star-Allele Caller for Pharmacogenomics. Genome Res..

[33] Numanagić I., Malikić S., Ford M., Qin X., Toji L., Radovich M., Skaar T.C., Pratt V.M., Berger B., Scherer S. (2018). Allelic Decomposition and Exact Genotyping of Highly Polymorphic and Structurally Variant Genes. Nat. Commun..

[34] Sangkuhl K., Whirl-Carrillo M., Whaley R.M., Woon M., Lavertu A., Altman R.B., Carter L., Verma A., Ritchie M.D., Klein T.E. (2020). Pharmacogenomics Clinical Annotation Tool (Pharm CAT). Clin. Pharmacol. Ther..

[35] Lee S., Shin J.-Y., Kwon N.-J., Kim C., Seo J.-S. (2022). ClinPharmSeq: A Targeted Sequencing Panel for Clinical Pharmacogenetics Implementation. PLoS ONE.

[36] Lee S., Wheeler M.M., Thummel K.E., Nickerson D.A. (2019). Calling Star Alleles with Stargazer in 28 Pharmacogenes with Whole Genome Sequences. Clin. Pharma Ther..

[37] Lee S., Wheeler M.M., Patterson K., McGee S., Dalton R., Woodahl E.L., Gaedigk A., Thummel K.E., Nickerson D.A. (2019). Stargazer: A Software Tool for Calling Star Alleles from next-Generation Sequencing Data Using CYP2D6 as a Model. Genet. Med..

[38] Gordon A.S., Fulton R.S., Qin X., Mardis E.R., Nickerson D.A., Scherer S. (2016). PGRNseq: A Targeted Capture Sequencing Panel for Pharmacogenetic Research and Implementation. Pharmacogenet. Genom..

[39] McLaren W., Gil L., Hunt S.E., Riat H.S., Ritchie G.R.S., Thormann A., Flicek P., Cunningham F. (2016). The Ensembl Variant Effect Predictor. Genome Biol..

[40] Lek M., Karczewski K.J., Minikel E.V., Samocha K.E., Banks E., Fennell T., O’Donnell-Luria A.H., Ware J.S., Hill A.J., Exome Aggregation Consortium (2016). Analysis of Protein-Coding Genetic Variation in 60,706 Humans. Nature.

[41] Adzhubei I.A., Schmidt S., Peshkin L., Ramensky V.E., Gerasimova A., Bork P., Kondrashov A.S., Sunyaev S.R. (2010). A Method and Server for Predicting Damaging Missense Mutations. Nat. Methods.

[42] Tordai H., Torres O., Csepi M., Padányi R., Lukács G.L., Hegedűs T. (2024). Analysis of AlphaMissense Data in Different Protein Groups and Structural Context. Sci. Data.

[43] De Sainte Agathe J.-M., Filser M., Isidor B., Besnard T., Gueguen P., Perrin A., Van Goethem C., Verebi C., Masingue M., Rendu J. (2023). SpliceAI-Visual: A Free Online Tool to Improve SpliceAI Splicing Variant Interpretation. Hum. Genom..

[44] Machiela M.J., Chanock S.J. (2015). LDlink: A Web-Based Application for Exploring Population-Specific Haplotype Structure and Linking Correlated Alleles of Possible Functional Variants. Bioinformatics.

