# Supplementary material for: Population-Scale Pharmacogenomic Profiling in the Western Balkan Populations
Source: Pharmaceuticals (Basel). 2026 Sep 26;19(10):1526. doi: 10.3390/ph19101526 (PMC13708261; doi:10.3390/ph19101526)
Supplement: Supplementary file 1 [file pharmaceuticals-19-01526-s001.zip › Supplementary PGH_revised.pdf]

Table S1. Subject characteristics (n=250)

Characteristics n					
**Country**	Bosnia and Herzegovina	Croatia	Montenegro	North Macedonia	Serbia
Sampling site	University Clinical Centre of the Republic of Srpska, Banja Luka	Department of Pediatrics, Clinical Hospital Centre Rijeka, Rijeka	Center for Medical Genetics and Immunology, Clinical Center of Montenegro, Podgorica	University Clinic for Pediatrics, Faculty of Medicine, Skopje	Institute of Molecular Genetics and Genetic Engineering, University of Belgrade, Belgrade
Number of samples	50	50	50	50	50
**Age, years**					
Median	55	36	40	37	41.5
Interquartile range	46-65	29.25-45	31-50.25	33-45	36-46.75
**Gender**					
Male	25	28	22	25	25
Female	25	22	28	25	25

Table S2. Comparison of genotype calls across pharmacogenomic annotation tools (Aldy, PyPGx, PharmCAT and Stargazer) and identified sources of discordance.

	**Tool used for genotyping**				
	**Aldy4**	**PyPGx**	**PharmCAT**	**Stargazer**	**Problem**
**Gene**	**Concordance (%)**				
ABCG2	100				
CACNA1S	100				
CFTR	98.8				All samples were reported as reference by PharmCATx whereas variants were detected by Aldy4 and PyPGx
COMT	NA				Different allele definitions
CYP1A1	100				
CYP1A2	100				
CYP2A6	95.2				Structural variant complexity
CYP2A13	100				
CYP2B6	90				Phasing/ distant variants
CYP2C8	100				
CYP2C9	100				
CYP2C19	94.6				Only Aldy4 took into consideration c.276G>A, whereas it was not reported by PharmCAT or PyPGx
CYP2D6	82.8			59.2	Structural variant complexity

CYP2F1	96	Different allele definitions
CYP2J2	100	
CYP2R1	100	
CYP2S1	100	
CYP2W1	100	
CYP3A4	99.6	Phasing / distant variants
CYP3A5	98.8	For two samples, Aldy4 reported the diplotype as *2/*3, whereas PyPGx and PharmCAT assigned *3/*3. The correct diplotypes were *3/*3
CYP3A7	100	
CYP3A43	89.2	PyPGx did not report *2B alleles detected by Aldy
CYP4F2	84.8	False *17 detection by PyPGx and PharmCAT
DPYD	71.6	Different allele definitions, c.85T>C (*9A) and c.496A>G wrong calls by PyPGx
G6PD	100	
GSTM1	100	
GSTP1	99.6	For one sample, Aldy4 reported the diplotype as *A/*B, whereas PyPGx assigned *A/*C. The correct diplotype was *A/*B
NAT1	67.2	PyPGx did not report several alleles detected by Aldy, including *10, *3, and *26
NAT2	93.2	PyPGx did not report several alleles detected by Aldy, including *12 and *16
NUDT15	100	
RYR1	72.4	All samples were reported as reference by PyPGx, whereas variants were detected by Aldy and PharmCAT
SLCO1B1	90	PharmCAT occasionally returned multiple possible diplotype assignments for the same sample, resulting in ambiguous genotype interpretation
TPMT	100	
UGT1A1	84	Phasing / distant variants
UGT2B7	100 (*2)	
VKORC1	100 (rs9923231)	
